# 1488. The Performance of a Multidimensional Frailty Index in Comparison to the Frailty Phenotype to Assess Frailty in an Urban HIV clinic.

**DOI:** 10.1093/ofid/ofad500.1323

**Published:** 2023-11-27

**Authors:** Uzoamaka A Eke, Kareshma Mohanty, Sarah Schmalzle, Nicole Viviano, Jennifer Hoffman, Pandit Neha, Robyn Palmeiro, Judith Lee, Kristen A Stafford, Ann Gruber-Baldini

**Affiliations:** University of Maryland School of Medicine, Baltimore, Maryland; University of MD, Baltimore, Maryland; University of Maryland School of Medicine, Baltimore, Maryland; University of MD, Baltimore, Maryland; Institute of Human Virology, University of MD, baltimore, Maryland; University of Maryland School of Pharmacy, Baltimore, Maryland; Institute of Human Virology, University of MD, baltimore, Maryland; Institute of Human Virology, University of MD, baltimore, Maryland; University of Maryland, Baltimore, MD; Division of Gerontology, Department of Epidemiology and Public Health, University of Maryland School of Medicine, Baltimore, Maryland

## Abstract

**Background:**

Frailty is increasingly recognized in people living with HIV (PLWH), but optimal diagnostics are yet to be determined. Frailty indices (FI) represent accumulation of health deficits over time and have been shown to correlate better with mortality and adverse effects of aging than the phenotypic description of frailty or chronological age. This study compares a new FI to the Fried frailty phenotype (FP) to diagnose frailty and determine factors associated with frailty.

**Methods:**

The Strengthening Therapeutic Resources in Older Adults Aging with HIV (STRONG) study incorporated standardized geriatric assessments in an adult HIV clinic in PLWH ≥50 years. FP scores of 0, 1-2, and ≥3 out of 5 criteria represented robust, pre-frail and frail, respectively. A 40-variable FI construct comprising demographic, clinical, and laboratory values was derived to calculate the FI as a fraction of the deficits present in each patient to the total variables that comprised the index. FI scores of ≤0.15, >0.15-0.4 and >0.4 were categorized as robust, pre-frail and frail respectively. FI and FP were compared with respect to frailty prevalence. Multivariate logistic regression models adjusted for age and sex were used to examine the association between FI frailty and each of the following factors: multimorbidity, falls, poor cognition history, polypharmacy (≥5 medications) and HIV duration.

**Results:**

The 165 participants were mostly black (94%) and male (56%), with median age 59 years (IQR 55-63). 78% were virally suppressed (HIV viral load ≤40) with median CD4 count 606 cells/μl (IQR 393-873). 70% had multimorbidity (≥2 comorbidities), 38% falls, 25% poor cognition history, 24% polypharmacy and 32% < high school education. Using FP, 65% were prefrail, 2% frail, and 33% robust. Using FI, 67% were prefrail, 26% frail and 7% robust (range 0.08-0.57; mean 0.34 ±0.11). For FP categorized as robust, prefrail and frail, the mean FI was 0.31±0.1, 0.35±0.11 and 0.38±0.08 respectively (P=0.06). Poor cognition (OR 3.91, p=0.001), falls (OR 4.49, p < 0.001) and multimorbidity (OR 5.09, p=0.004) were associated with FI frailty, after adjusting for age and sex.

**Conclusion:**

The majority of our PLWH are pre-frail or frail. The FI identified more patients as frail and had significant clinical associations compared to FP.

**Disclosures:**

**All Authors**: No reported disclosures

